# NCL1, a highly selective lysine-specific demethylase 1 inhibitor, suppresses prostate cancer without adverse effect

**DOI:** 10.18632/oncotarget.3067

**Published:** 2014-12-26

**Authors:** Toshiki Etani, Takayoshi Suzuki, Taku Naiki, Aya Naiki-Ito, Ryosuke Ando, Keitaro Iida, Noriyasu Kawai, Keiichi Tozawa, Naoki Miyata, Kenjiro Kohri, Satoru Takahashi

**Affiliations:** ^1^ Department of Nephro-Urology, Nagoya City University, Graduate School of Medical Sciences, Nagoya, Japan; ^2^ Department of Chemistry, Kyoto Prefectural University of Medicine, Graduate School of Medical Science, Kyoto, Japan; ^3^ Department of Experimental Pathology and Tumor Biology, Nagoya City University, Graduate School of Medical Sciences, Nagoya, Japan; ^4^ Institute of Drug Discovery Science, Nagoya City University, Graduate School of Pharmaceutical Sciences, Nagoya, Japan

**Keywords:** LSD1, epigenetics, prostate cancer, autophagy

## Abstract

Herein, we investigated therapeutic potential of a novel histone lysine demethylase 1 (LSD1) inhibitor, NCL1, in prostate cancer. Hormone-sensitive prostate cancer cells, (LNCaP) and castration resistant cancer cells (PC3 and PCai1) were treated with NCL1, and LSD1 expression and cell viability were assessed. Prostate cancer cells showed strong LSD1 expression, and cell viability was decreased by NCL1. ChIP analysis showed that NCL1 induced H3K9me2 accumulation at the promoters of androgen-responsive genes. NCL1 also induced G1 cell cycle arrest and apoptosis. In addition, autophagosomes and autolysosomes were induced by NCL1 treatment in LNCaP. Furthermore, LC3-II expression was significantly increased by NCL1 and chloroquine. In mice injected subcutaneously with PCai1 and intraperitoneally with NCL1, tumor volume was reduced with no adverse effects in NCL1-treated mice. Finally, LSD1 expression in human cancer specimens was significantly higher than that in normal prostate glands. In conclusion, NCL1 effectively suppressed prostate cancer growth without adverse events. We suggest that NCL1 is a potential therapeutic agent for hormone-resistant prostate cancer.

## INTRODUCTION

Prostate cancer is one of the most frequently diagnosed cancers in the Western world. There are many therapeutic options against localized prostate cancer. However, in advanced cancers, most tumors ultimately relapse after a period of initial response to therapy and progress to metastatic cancer [1], for which effective therapeutic procedures are extremely limited. Administration of docetaxel has been established as a new standard of chemotherapy for those patients [2-4]. However, it is not curative, and optimal timing of administration remains controversial. Therefore, understanding the mechanisms of prostate cancer development and finding new therapeutic options are clinically necessary.

Prostate cancer development is a multistep process that results from changes in genetic mechanisms. Recently, it has become apparent that not only genetic mutations but also epigenetic alterations lead to the activation of oncogenes and the loss of function of tumor suppressor genes. Since epigenetic abnormalities in DNA methylation patterns as well as histone modifications are potentially reversible in contrast to gene mutations, much effort has been directed toward understanding the mechanisms of epigenetic aberration to develop new therapy. Indeed, inhibitors of histone deacetylases, such as vorinostat and romidepsin, and DNA methyltransferases, such as azacytidine have been clinically approved for treatment of certain types of cancers [5-8], and there were some reports about inhibitors of histone deacetylases for prostate cancer in basic research [9, 10].

The N-terminal tails of histones are subjected to several types of post-translational modifications, including acetylation, methylation, and phosphorylation [11]. The combination of these modifications determines chromatin structure and transcriptional activation or repression of genes. These modifications are regulated by two classes of enzymes with opposing activities: histone methyltransferases and histone lysine demethylases (LSD). LSD1 is the first of this type of enzyme that was found to belong to the flavin adenine dinucleotide dependent enzyme family. LSD1 functions as a histone demethylase that removes mono- and dimethyl, but not trimethyl marks from either lysine 4 or 9 of histone H3 (H3K4 and H3K9, respectively) [12].

Previous studies showed that the expression of LSD1 positively correlates with the malignancy of various organs [13-17]. The members of the amine oxidase family also impair the activity of LSD1. However, many of these amine oxidase inhibitors including clorgyline, pargyline, tranylcypromine (PCPA), polyamines and derivatives thereof do not selectively target LSD1 [13, 18], which limits their use as therapeutics owing to potential side effects. In the previous study, we developed a novel and selective LSD1 inhibitor called NCL1 (N-[(1S)-3-[3-(trans-2-Aminocyclopropyl)phenoxy]-1-(benzylcarbamoyl)propyl] benzamide) by combining protein structure similarity clustering and *in vitro* screening [19]. NCL1 impairs LSD1 demethylase activity and blocks cell proliferation in prostate cancer, glioma, and breast cancer cell lines [20, 21]. However no report has described the effects of NCL1 treatment using an *in vivo* model for prostate cancer.

In this study, we examined the LSD1 status in prostate cancer cell lines and human prostatectomy specimen. In addition, we tested the therapeutic significance of NCL1 in prostate cancer cells *in vitro* and in an *in vivo* subcutaneous model. Furthermore, we investigated the pharmacological mechanism of NCL1 using ChIP assay, flow cytometry, and western blotting analyses. We are the first to find that treatment with NCL1 to inhibit LSD1 induced cell death through regulation of autophagy in prostate cancer.

## RESULTS

### LSD1 expression in prostate cancer cell lines and suppression of prostate cancer cell proliferation by NCL1

**Figure 1:**
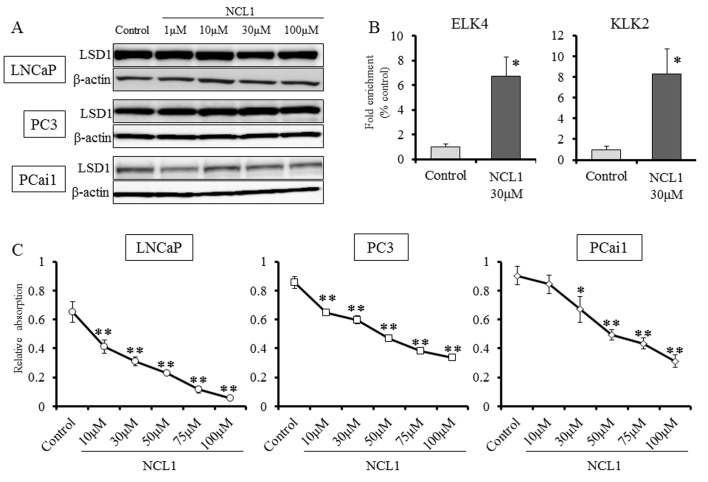
Inhibition of LSD1 functions reduced the proliferation of prostate cancer cell lines A, Western blot analysis of PCai1, PC3, and LNCaP cells for LSD1. All prostate cancer cell lines expressed LSD1. β-actin was used as internal loading control. B, ChIP analysis using an H3K9me2 antibody showed that NCL1 induced accumulation of H3K9me2 at the promoter regions of the AR target genes, *KLK2* and *EKL4*. C, PCai1, PC3, and LNCaP cells were seeded in 96-well plates (5 × 10^3^ cells/well), treated with vehicle or NCL1, and subjected to the WST-8 assay at 48h. NCL1 treatment reduced cell viability of all prostate cell lines in a dose-dependent manner. Mean ± SD; *P<0.05, **P<0.001.

We first examined the status of LSD1 in prostate cancer cells, and found by western blotting analysis that the protein expression level of LSD1 was not changed after NCL1 treatment (Fig. 1A). To determine whether LSD1 inhibition influences gene specific methylation status, LNCaP cells treated with NCL1 were subjected to ChIP assay. Consistent with our previous report, NCL1 specifically impaired the demethylation of H3K9me2 at the androgen-response elements containing promoters of ETS domain-containing protein 4 (*ELK4*) and kallikrein 2 (*KLK2*) genes (Fig. 1B). Cell proliferation assay revealed that prostate cancer cell proliferation following NCL1 treatment was significantly decreased in a dose-dependent manner in all cancer cell lines (Fig. 1C). These results indicated that NCL1 has the ability to attenuate demethylation of H3K9me2 to regulate androgen responsive genes. On the other hand, the proliferation rate after NCL1 treatment was also significantly reduced in a dose-dependent manner in PrEC, a normal prostate cell line that expresses LSD1 and androgen receptor (AR). However, the effect in PrEC was much weaker than in prostate cancer cell lines (Fig. 5A, 5B). Therefore, NCL1 has the potential to suppress cell proliferation, with higher sensitivity in cancer cells as compared to normal cells.

### NCL1 inhibits LNCaP cell growth by apoptotic and growth arrest mechanisms

**Figure 2:**
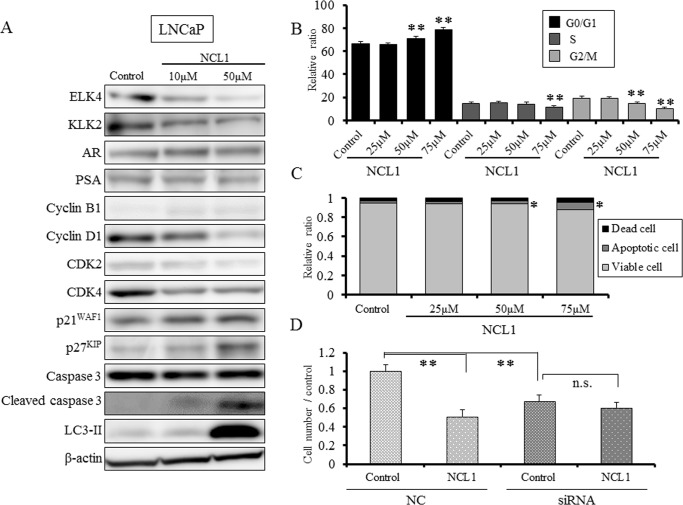
NCL1 treatment induced cell cycle arrest and apoptosis in LNCaP cells A, Western blotting analyses following NCL1 treatment of LNCaP cells. The expression of ELK4 and KLK2 were reduced, but those of AR and PSA were not changed. Treatment with NCL1 resulted in a marked reduction in the expression of cyclin D1 and elevation in cleaved caspase 3. Assessment of other proteins involved in cell cycle and apoptosis, demonstrated that CDK2 and CDK4 were decreased, and p21^WAF1^ and p27^KIP^ were increased by NCL1 treatment. β-actin was used as internal loading control. B, Guava^®^ cell cycle analysis of LNCaP cells. NCL1-treated LNCaP cells significantly accumulated in the G0/G1 phase in a dose-dependent manner. C, Guava^®^ apoptosis analysis of LNCaP cells. NCL1 induced apoptosis of LNCaP cells in a dose-dependent manner. D, LNCaP cells were seeded in 6-well plates (2 × 10^5^ cell/well) and transfected with LSD1 or negative control (NC) siRNA. Four days after transfection, 30 μM NCL1 or vehicle added. For monitoring growth inhibition, cells were trypsinized on day 7 after transfection, and then the cell numbers were counted. LSD1 siRNA treatment reduced cell viability of LNCaP cells, and NCL1 treatment did not affect the viability of LSD1 siRNA-transfected LNCaP cells. Mean ± SD; *P<0.05, **P<0.001.

To explore the underlying mechanisms of NCL1 induced growth inhibition, we evaluated the profiles of proteins involved in apoptosis and cell cycle after NCL1 treatment in LNCaP cells. Reflecting the attenuation of LSD1, the expression of ELK4 and KLK2 were reduced, but the expression of AR and prostate specific antigen (PSA) were not changed (Fig. 2A). Treatment with NCL1 resulted in a marked reduction in the expression of cyclin D1 and elevation in cleaved caspase 3 with no alteration in caspase 3 expression. Examination of cell cycle-related proteins showed that cyclin dependent kinase (CDK) 2 and CDK4 expression were decreased, while those of p21^WAF^ and p27^KIP^ were increased by NCL1 treatment (Fig. 2A). Therefore, cell cycle and viacount analyses by the Guava^®^ assay were performed. As a result, we found that NCL1 treatment of LNCaP cells led to significant accumulation in the G0/G1 phase (Fig. 2B) and induction of apoptosis (Fig. 2C) in a dose-dependent manner. These results suggested that selective attenuation of LSD1 using NCL1 inhibits cell proliferation by caspase dependent apoptosis and cell growth arrest in the G0/G1 phase in a cyclin D1-dependent manner. Next, to clarify the selective inhibitory effect of LSD1, we knocked down LSD1 using siRNA in LNCaP cells. Attenuation of LSD1 by siRNA caused significant reduction of cell growth compared with negative control (NC) transfection. However, treatment with NCL1 induced no further inhibitory effects on LSD1-silenced LNCaP, while NCL1 significantly inhibited cell growth in NC-transfected LNCaP (Fig. 2D). These results indicated that the anti-tumor effect of NCL1 was specific for LSD1 function.

### NCL1 potentially regulates autophagy to induce cell death in LNCaP cells

**Figure 3:**
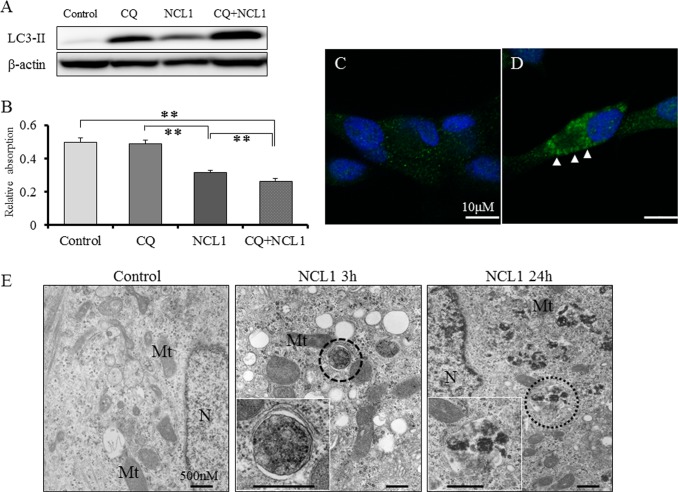
NCL1 treatment induced autophagy in LNCaP A, Detection of autophagy by western blot analysis for LC3-II. Expression of LC3-II was elevated in NCL1-treated LNCaP cells. CQ synergized with NCL1 which resulted in enhanced elevation of LC3-II expression. β-actin was used as internal loading control. B, Cells were treated with 50 μM NCL1 and/or 40 μM CQ for 24 h. WST-8 assay, in which dye absorption rate is positively correlated with cell viability, revealed that a combination of NCL1 and CQ significantly decreased cell growth. Mean ± SD; **P<0.001. C, D, Immunofluorescence for LC3-II was performed at 24h after treatment. Expression of LC3-II in the cytoplasm was higher in the CQ + NCL1 group (D) than in controls (C). Blue staining denotes DAPI-labeled nuclei. E, Cells were treated with 50 μM NCL1 for 3h or 24h. By TEM, autophagosomes were observed (marked by broken line circle) in LNCaP cells treated for 3h with NCL1, and autolysosomes were observed (marked by dotted line circle) in LNCaP cells treated for 24h with NCL1. N; nucleus, Mt; mitochondrion

As conversion of LC3-I to LC3-II and formation of LC3 puncta have been generally used as indicators of autophagy [22], we employed them to determine whether NCL1 treatment induced autophagy in prostate cancer cells. By western blotting analyses, we found that NCL1 induced an increase of LC3-II levels in LNCaP cells (Fig. 3A). Therefore, to confirm the contribution of NCL1 to autophagy, we inhibited the autophagic flux with chloroquine (CQ), which raises the pH within the lumen of lysosomes and/or autolysosomes thereby compromising autophagic degradation. It was revealed by western blotting analysis that CQ induced LC3-II expression, and addition of NCL1 led to a further accumulation of LC3-II. By WST assay, CQ alone didn't have an effect on LNCaP cell viability after treatment for 24h, but CQ enhanced the efficacy of NCL1-induced cell growth inhibition in LNCaP cells (Fig. 3B). Immunofluorescent staining further revealed that the cytoplasmic expression of LC3-II was significantly increased in NCL1- and CQ-treated cells as compared to control (Fig. 3C, 3D). By TEM, autophagosomes were clearly observed 3h after treatment with NCL1, and autolysosomes were strongly observed 24h after treatment (Fig. 3E). These morphological features clearly reflect the classical characteristics of autophagy [23-25]. These results suggest that NCL1 has the potential to induce cancer cell death through the regulation of autophagy potentiate in addition to the regulation of apoptotic anticancer pathway.

### *In vivo* regulation of tumorigenesis by NCL1

To evaluate the roles of NCL1 in tumor progression *in vivo*, PCai1 cells were transplanted subcutaneously in nude mice, which were subsequently treated with 0.5 mg/kg or 1.0 mg/kg of NCL1. As a control, a group of mice were treated with 10 mg/kg tranylcypromine (PCPA), a broad spectrum LSD1 inhibitor. Tumor growth was significantly inhibited in the 1.0 mg/kg NCL1 treated group as compared to the vehicle controls (Fig. 6A). NCL1 did not affect other organs and body weight. There were no differences in the relative weight of organs and blood examination between the 4 groups, except for the increased relative liver and kidney weights in the PCPA group (Table 1A, 1B). By immunohistochemical analysis, the representative expression patterns of LSD1 in major organs, including normal prostate, testis, kidney, lung, liver, and brain, in the vehicle control group are demonstrated in Fig. 5C-N. NCL1 did not induce any tissue damage in these organs (data not shown). Microscopically, vacuolization was increased in a dose-dependent manner in the NCL1 treated groups compared with the vehicle control group (Fig. 6D, 6E). To verify the results of the *in vitro* study, the mechanisms of attenuation of tumor growth by NCL1 *in vivo* were examined using TUNEL assay. Administration of NCL1 significantly increased the appearance of TUNEL-positive cells, and thus apoptosis, compared with vehicle control and PCPA in a dose-dependent manner (Fig. 6B, 6F, 6G). In addition, we examined tumor vascularity using immunohistochemistry of CD31. We found that CD31-positive vessels were significantly decreased in NCL1 treated tumors (Fig. 6C, 6H, 6I). In addition, western blotting analysis revealed that the expression of LC3- II was increased in the NCL1 treatment group as compared to the vehicle controls (Fig. 6J). These results suggested that NCL1 decreased tumor vascularity, and induced cell death through the regulation of apoptosis and autophagy both *in vitro* and *in vivo*.

**Table 1:** NCL1 treatment had no effect on relative organ weight and results of blood examination **A**, The results of relative organ weight at experiment termination. BW: Body weight; PCPA: tranylcypromine. There were no differences in the relative weight of organs between the 4 groups, except for the increased relative liver and kidney weights in the PCPA group. **B**, The results of blood examination at experiment termination. There were no differences in the blood examination between the 4 groups.

A								
		No. of mice	BW (g)	Liver (%)	R-kidney (%)	L-kidney (%)	R-testis (%)	L-testis (%)
control		10	27.5 ± 1.6	4.63 ± 0.19	0.91 ± 0.03	0.91 ± 0.03	0.83 ± 0.05	0.80 ± 0.04
PCPA 10mg/kg		5	28.0 ± 1.0	5.21 ± 0.31*	1.30 ± 0.09*	1.30 ± 0.13*	0.82 ± 0.09	0.81 ± 0.04
NCL1	0.5mg/kg	10	27.8 ± 1.0	4.58 ± 0.12	0.93 ± 0.05	0.92 ± 0.05	0.84 ± 0.06	0.82 ± 0.05
	1.0mg/kg	10	27.8 ± 1.3	4.51 ± 0.20	0.92 ± 0.04	0.91 ± 0.04	0.83 ± 0.05	0.83 ± 0.05

B				
	control	PCPA 10mg/kg	NCL1	
			0.5mg/kg	1.0mg/kg
AST	42.5 ± 8.1	55.2 ± 9.0	61.3 ± 12.0	51.0 ± 6.2
ALT	22.5 ± 5.6	22.6 ± 2.1	27.0 ± 6.4	23.3 ± 2.9
ALP	194.0 ± 14.8	212.8 ± 15.0	169.7 ± 4.8	206.3 ± 17.6
T-Bil	0.10 ± 0.00	0.12 ± 0.04	0.10 ± 0.00	0.10 ± 0.00
T-Chol	83.3 ± 4.1	105.4 ± 6.0	95.7 ± 4.9	99.3 ± 2.2
Crea	0.20 ± 0.03	0.10 ± 0.01	0.10 ± 0.02	0.09 ± 0.01
BUN	24.3 ± 2.5	21.7 ± 1.9	20.4 ± 1.7	21.2 ± 1.7
Na	154.3 ± 1.5	150.4 ± 0.8	145.3 ± 2.5	147.5 ± 0.5
K	5.6 ± 0.4	4.7 ± 0.3	5.7 ± 0.5	5.5 ± 0.4
Cl	115.5 ± 1.7	113.2 ± 1.3	112.0 ± 1.6	113.3 ± 0.8
Ca	8.9 ± 0.2	8.8 ± 0.2	8.4 ± 0.1	8.2 ± 0.3

PCPA: tranylcypromine; AST: aspartate aminotransferase; ALT: alanine aminotransferase; ALP: alkaline phosphatase; T-Bil: total bilirubin; T-Chol: total cholesterol; Crea: creatinine; BUN: blood urea nitrogen; Na: sodium; K: potassium; Cl: chloride; Ca: calcium.

Mean ± SD;

* P<0.05; n = 5 (PCPA group), 10 (control, NCL1 groups).

**Figure 4:**
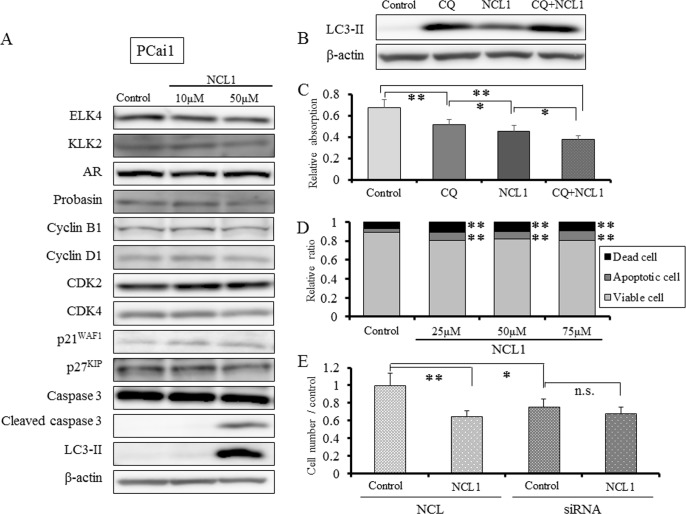
NCL1 treatment induced apoptosis and autophagy in PCai1 cells A, Western blotting analyses following NCL1 treatment of PCai1 cells. Treatment with NCL1 resulted in elevation in cleaved caspase 3. Assessment of proteins involved in the cell cycle, such as CDKs and cyclins, demonstrated no change by NCL1 treatment. β-actin was used as internal loading control. B, Detection of autophagy by western blot analysis for LC3-II. Expression of LC3-II was elevated in NCL1-treated PCai1 cells. CQ synergized with NCL1 which resulted in enhanced elevation of LC3-II expression. β-actin was used as internal loading control. C, PCai1 cells were treated with 50 μM NCL1 and/or 40 μM CQ for 24 h. WST-8 assay, in which dye absorption rate is positively correlated with cell viability, revealed that a combination of NCL1 and CQ significantly decreased cell growth. Mean ± SD; *P<0.05, **P<0.001. D, Guava^®^ apoptosis analysis of PCai1 cells. NCL1 induced apoptosis of PCai1 cells. E, PCai1 cells were seeded in 6-well plates (5×10^4^ cell/well) and transfected with LSD1 or negative control (NC) siRNA. Four days after transfection, 30 μM NCL1 or vehicle added. For monitoring growth inhibition, cells were trypsinized on day 7 after transfection, and then the cell numbers were counted. LSD1 siRNA treatment reduced cell viability of PCai1 cells, NCL1 treatment did not affect viability of LSD1 siRNA-transfected PCai1 cells. Mean ± SD; *P<0.05, **P<0.001.

### LSD1 is overexpressed in prostate cancer

To determine the status of LSD1 in human prostate cancer, LSD1 expression was examined by immunohistochemistry, and the staining intensity was scored using prostate cancer tissue arrays that contain tumors with different Gleason patterns as well as normal prostate tissues. Our findings demonstrated that LSD1 expression was significantly higher in tumor cells of prostate cancer tissues than in the luminal cells of normal prostate glands, and its expression level in Gleason pattern 5 tumors was slightly higher than those of other Gleason patterns, although the differences were not significant (Fig. 7A-E).

**Figure 5:**
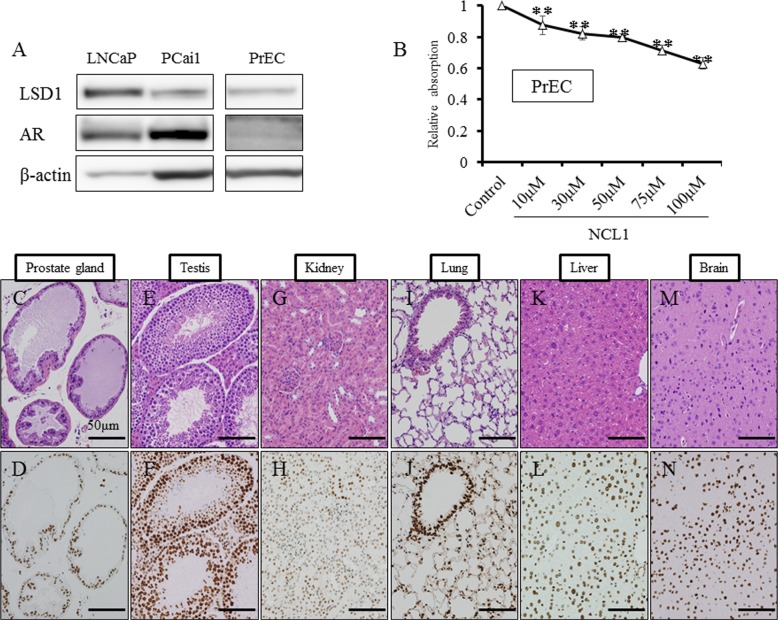
NCL1 treatment in normal prostate cell line, PrEC, and immunohistochemical analyses of LSD1 in mouse tissue A, Western blot analysis of LNCaP, PCai1 and PrEC cells for LSD1 and AR. PrEC expressed LSD1 and AR. β-actin was used as internal loading control. B, PrEC cells were seeded in 96-well plates (5 × 10^3^ cells/well), treated with vehicle or NCL1, and subjected to the WST-8 assay at 48h. The proliferation rate after NCL1 treatment was significantly reduced in a dose dependent manner. However, the effect in PrEC was weaker than in other prostate cancer cell lines. Mean ± SD; **P<0.001. C-N, HE staining in normal prostate glands (C), testis (E), kidney (G), lung (I), liver (K) and brain (M), and immunohistochemistry for LSD1 in normal prostate glands (D), testis (F), kidney (H), lung (J), liver (L) and brain (N) of the vehicle control group. Nuclei were counterstained with hematoxylin. LSD1 was differentially expressed in various organs.

**Figure 6:**
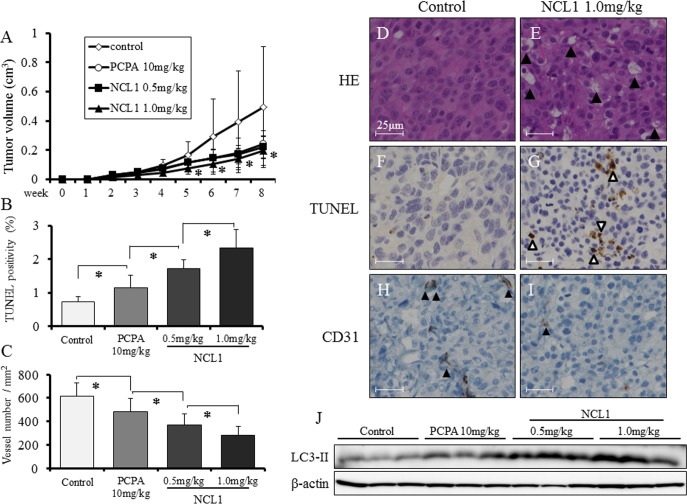
NCL1 treatment reduced subcutaneous prostate cancer xenograft tumor growth and induced apoptosis and autophagy *in vivo* A, Tumor growth was significantly inhibited in mice treated with 1.0 mg/kg NCL1 as compared to vehicle controls. B, TUNEL assay was performed in NCL1-treated and control mice, and quantified as mean TUNEL labeling percentage based on at least 5 randomly selected high-power microscope fields per individual. C, Immunohistochemistry for CD31. Positivity was quantified as mean number of vessels/mm^2^ based on at least 5 randomly selected high-power microsope fields per individual. D, E, HE staining in subcutaneous tumors from vehicle control (D) and 1.0 mg/kg NCL1-treated (E) mice. Vacuolation was increased in the NCL1-treated group compared with the controls. F, G, TUNEL staining for apoptosis in subcutaneous tumors from vehicle control (F) and 1.0 mg/kg NCL1-treated (G) mice. Triangles indicate TUNEL-positive cells. H, I, Representative immunohistochemical images of CD31 in subcutaneous tumors from control (H) and 1.0 mg/kg NCL1-treated (I) mice. Triangles indicate CD31-positive cells. J, Proteins were extracted from subcutaneous tumors and western blotting was performed. Expression of LC3-II in NCL1-treated tissues was higher relative to those in PCPA and vehicle controls. Nuclei were counterstained with hematoxylin. Mean ± SD; *P<0.05.

**Figure 7:**
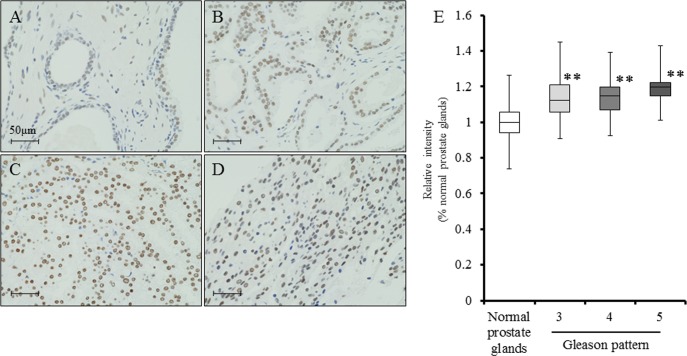
LSD1 expression is elevated in prostate cancer A-D, Immunohistochemistry for LSD1 in normal prostate glands (A), Gleason pattern 3 adenocarcinoma (B), Gleason pattern 4 adenocarcinoma (C), and Gleason pattern 5 adenocarcinoma (D). Nuclei were counterstained with hematoxylin. E, By tissue array analysis, LSD1 expression was significantly higher in prostate cancer cells of prostate cancer tissues than in the luminal cells of normal prostate glands, and expression levels of Gleason pattern 5 tumor was slightly higher than those of other Gleason pattern, but not significant. Nuclei were counterstained with hematoxylin. Mean ± SD; **P<0.001.

## DISCUSSION

In the present study, we tested the hypothesis that deregulation of LSD1 represses prostate cancer progression and evaluated the therapeutic effect of targeting this molecule using a novel selective LSD1 inhibitor, NCL1, originally developed at our institute [19]. Inhibition of LSD1 by NCL1 resulted in an increase in promoter-specific H3K9me2 modifications (Fig. 1B), and induced decreases in the protein expression of KLK2 and ELK4 (Fig. 2A). NCL1 not only had significant growth inhibitory properties *in vitro* (Fig. 1C), but it also attenuated tumor growth *in vivo* at extremely low concentration levels as compared to previous LSD1 inhibitors (Fig. 6A). In addition, side effects were not observed at all by both general condition and blood examination (Table 1). Therefore, we demonstrated the potential safety and utility of NCL1 as a therapeutic option. This is the first report to test the therapeutic potential of NCL1 *in vivo* by using a prostate cancer model.

LSD1 is involved in the regulation of broad gene expression programs in many malignancies [13-17]. We observed high protein expression of LSD1 in prostate cancer cells (Fig 1A, 5A), and the inhibition of LSD1 activity using NCL1 reduced cell proliferation *in vitro* (Fig. 1C, 2D, 4E). It has been reported that LSD1 inhibitors preferentially kill transformed or cancer cells in both cell culture and animal models [15, 19-21], however, the mechanisms by which LSD1 inhibitors induce cell death are not well understood. In this report, we found that NCL1 induced apoptosis both *in vitro* and *in vivo* (Fig. 2A, 2C, 4A, 4D, 6B, 6F, 6G). Cell death occurs through apoptosis or non-apoptosis, however, a lack of a strict definition for apoptosis often lead to controversy. How the cells die is determined whether they are apoptosis-reluctant or apoptosis-prone [26, 27]. Recently, autophagy, an alternative pathway of programmed cell death to apoptosis, plays an important role in cell death induced by HDAC inhibitor [25], but there has been no report on autophagy stimulated by LSD1 inhibition in prostate cancer cells. Cell death induced by apoptosis and autophagy frequently occur in parallel, however caspases are not required for autophagic cell death, and autophagic activity was found to be suppressed in malignant tumors [23, 24, 28]. To date, the most convincing and standard method to detect autophagy is to examine the ultrastructure of cells by TEM [24]. We investigated drug-induced autophagy in LNCaP cells using TEM and western blotting to detect LC3-II expression, which is a non-specific autophagic marker (Fig. 3A-E). Our results demonstrated that NCL1 induced autophagy in LNCaP cells in a concentration dependent manner, which correlated well with their cytotoxic effects. It is generally accepted that autophagy exerts a tumor suppression function [24, 29] as a pro-death process, at least in cancer cells with defective apoptosis machinery [30]. When autophagy was inhibited by CQ, the anti-tumor effect of NCL1 was reinforced. So, this suggests that autophagy in cancer cells is a protective reaction to anti-tumor agents, and a disrupted autophagy pathway may enhance the cytotoxicity of LSD1 inhibitor in prostate cancer cells.

LSD1 plays a key role in many physiological functions, and recent studies suggested that knock down of LSD1 reduces cell growth by affecting expression of several genes involved in proliferation including p21, cyclin A2, and v-erb-b2 avian erythroblastic leukemia viral oncogene homolog 2, and promotes cell cycle arrest [17, 31-33]. In our study, we found that NCL1 induced cyclin D1 dependent cell cycle arrest *in vitro* (Fig. 2A, 2B). The CDK inhibitors, p21^WAF1^ and p27^KIP^, negatively modulate cell cycle progression by inhibiting the activity of cyclin/CDK complexes and block DNA replication by binding to the proliferating cell nuclear antigen [34-37]. The most recognized function of CDKs is phosphorylation of retinoblastoma (Rb) that is in a complex with the transcription factor E2F [35, 38]. Rb hyperphosphorylation in late G1 phase disrupts its association with various E2F family members, allowing the coordinated transcription of numerous genes whose products are necessary for DNA synthesis. In the present study, NCL1 significantly increased p21^WAF1^ and p27^KIP^ protein expression, and decreased CDK2, CDK4 and cyclin D1 in LNCaP cells in concentrations within their cytotoxic ranges. These effects would result in a decrease of E2F or phosphorylated Rb which is a downstream target of this pathway. In addition, interestingly, the inhibitory mechanisms of cancer growth by NCL1 appeared to be related not only to the direct effects on cell proliferation but also angiogenesis, as confirmed by a reduction in CD 31-positive vessels *in vivo* (Fig. 6C, 6H, 6I).

WST assay revealed that the 50% attenuation rate of cell growth in LNCaP was less than 30 μM NCL1, while that of PCai1 and PC3 was about 50 μM. LNCaP is a hormone naïve prostate cancer cell line, but PC3 and PCai1 are castration resistant prostate cancer cell lines. Therefore, the reason for this altered response may be partly due to the more aggressive and resistant phenotype of the latter cells. In PrEC cells, the 50% attenuation rate of cell growth was over 100 μM NCL1, which indicated that NCL1 was substantially less effective in normal prostate cells. To examine the effect of NCL1 in castration resistant cells, we performed the same *in vitro* analyses in PCai1 as in LNCaP. As seen in LNCaP, LSD1 siRNA treatment reduced cell viability of PCai1, and cell viability was not affected by NCL1 treatment in LSD1 siRNA-transfected PCai1 unlike in NC-transfected PCai1. The mechanism of growth inhibitory effect was via caspase dependent apoptosis and autophagy (Fig. 4A-E), but the cell cycle was not involved in the anti-tumor effect of NCL1 in PCai1 unlike in LNCaP (Fig. 4A).

For therapeutic application, validation of the therapeutic and the toxic efficiencies *in vivo* is required. Tumorigenesis of PCai1 cells *in vivo* was stronger than that of LNCaP, therefore, to demonstrate the effect of NCL1 in prostate cancer, we used PCai1 in the *in vivo* study. We found that NCL1 suppressed the proliferation of PCai1 xenografts by induction of apoptosis and autophagy (Fig. 6). No reports have described LSD1 expression in the major organs of mouse. Therefore, to understand the effect of NCL1 in mice, we performed immunohistochemical analyses in the *in vivo* study. The expression of LSD1 protein was highly valuable in the different mouse organs, ranging from weak to strong (Fig. 5C-N). However, regardless of the level of LSD1 expression, NCL1 had no toxic effect in not only normal prostate glands, but also other tissues (data not shown). There were some related reports suggested other treatments that modulate apoptosis/autophagy such as CQ enhanced the growth inhibition in prostate cancer [39-41]. These results suggest that NCL1 can be used as a novel curative drug for prostate cancer. Further, when used in combination with autophagy regulation drugs, NCL1 may be more effective in suppression of prostate cancer growth.

In two reports, high expression of LSD1 in prostate cancer was shown to be a predictive marker for aggressive tumor biology and tumor recurrence during therapy [13, 14]. Our immunohistochemical analyses using tissue arrays consisting of Japanese prostatectomy specimens also demonstrated that LSD1 expression was significantly higher in cancer cells of tumor tissues than in normal prostate luminal cells (Fig. 7A-D). The expression level of LSD1 in Gleason pattern 5 tumors tended to be higher than that in Gleason pattern 3/4 cases (Fig. 7E). In addition, cell growth of PCai1 cells, which are able to grow under castration-resistant conditions [42], was effectively suppressed by NCL1 both *in vitro* and *in vivo*. Therefore, NCL1 might have therapeutic potential for aggressive forms of human prostate cancer such as castration-resistant tumors. Further studies are needed to clearly test its *in vivo* potential in combination with hormonal therapy.

In summary, NCL1 effectively suppressed prostate cancer growth *in vitro*, and had strong efficacy without adverse events *in vivo* via regulation of apoptosis and autophagy. Further, human prostate cancers were found to harbor strong expression of LSD1. Therefore, our findings suggest that NCL1 is a novel potential therapeutic agent for castration resistant prostate cancer.

## METHODS

### Cell culture and treatments

The human prostate cancer cell lines, LNCaP and PC3, the human normal prostate cell line, PrEC, purchased from American Type Culture Collection (Manassas, VA), and an originally established castration resistant rat prostate cancer cell line, PCai1, were cultured as described previously [42, 43]. PCPA was purchased from Sigma-Aldrich Chemical Co (St. Louis, MO). NCL1 was synthesized as previously described [19].

LNCaP, PC3, PCai1, and PrEC cells were treated with vehicle containing DMSO that was equal in concentration to 100 μM NCL1 or with 1-100 μM NCL1 for 48h to determine its effect on cell growth. LNCaP and PCai1 cells were treated with 50 μM NCL1 and/or 40 μM CQ for 24h to assess the autophagic effects on cell proliferation.

All experiments were performed in triplicate.

### Cell proliferation assay

Cell proliferation rates were measured by using the WST-8 Cell Counting Kit (Wako, Osaka, Japan) in 96-well microplates. Prostate cancer cells were seeded in 96-well plates (5 × 10^3^ cells/well) in DMEM medium containing 10% FBS. After an overnight incubation, growth rate was determined by using the WST tetrazolium dye, which is reflected by the rate of dye absorption.

### Chromatin immunoprecipitation (ChIP) assay

LNCaP cells were cultivated for 210 min in the presence or absence of 30 μM NCL1 as indicated. Then, cells were cross-linked using 1% formaldehyde, and the chromatin was subjected to immunoprecipitation using an H3K9-me2 antibody (Cell Signaling Technology, Beverly, MA). Isotype-specific IgG was used as a control. Extracted DNA was dissolved in TE buffer and subjected to real-time PCR using *ELK4* and *KLK2* specific primers: *ELK4* F-GGGTGATGAACGAAGGCTTG, *ELK4* R-CTCCAGAGCAGACTTAGCTG; and *KLK2* F-ACCCCTGTTGCTGTTCATCCTG, *KLK2* R-CCGCCCTTGCCCTGTTGG.

### Western blotting analysis

Cells were lysed in SDS buffer, and 10 μl of protein was resolved in 12% polyacrylamide gels and transferred onto Hybond ECL membranes (GE Healthcare, Piscataway NJ). ELK4 (Abcam, Cambridge, UK), KLK2 (Santa Cruz Biotechnology, Santa Cruz, CA), cyclin B1 (Santa Cruz), cyclin D1 (Santa Cruz), CDK2 (Santa Cruz), CDK4 (Santa Cruz), caspase 3 (Cell Signaling), cleaved caspase 3 (Cell Signaling), prostate specific antigen (PSA) (Dako, Glostrup, Denmark), AR (Santa Cruz), p21^WAF1^(Cell Signaling), p27^KIP^ (Santa Cruz), and LC3-II (Abcam) antibodies were used to assess their protein expression levels. Beta actin expression was evaluated to confirm equal amount of protein loading using a monoclonal anti-beta-actin antibody (Sigma-Aldrich).

### Flow cytometry analysis

LNCaP cells (2 × 10^5^) were treated with NCL1 for 72h then cell suspensions were prepared and stained with Guava^®^ ViaCount reagent and propidium iodide according to the Guava^®^ Assay protocol (Guava Technologies, Hayward, CA). Apoptotic analysis and cell cycle phase distributions were determined on a Guava^®^ PCA Instrument using the CytoSoft Software.

### siRNA transfection *in vitro* and cell growth assay

Stealth Select RNAi targeting rat and human *LSD1* sequences were obtained (Sigma Aldrich). LNCaP (2 × 10^5^) and PCai1 (5 × 10^4^) cells were seeded in six-well plates and transfected with 30 nM siRNA using LipofectAMINE RNAiMAX Life Technologies, Carlsbad, CA) according to the manufacturer's protocol. Silencer negative control siRNA with no significant homology to any known rat and human genes was used as a negative control. The ability of siRNA to silence LSD1 protein expression was checked on the third day after transfection. Four days after transfection, 30μM NCL1 or vehicle was added. For monitoring growth inhibition, cells were trypsinized on day 7 after transfection, and then the cell numbers were counted.

### Transmission electron microscopy (TEM)

LNCaP cells were seeded in 6-well plates (3 × 10^5^ cells/well) in DMEM medium containing 10% FBS. After an overnight incubation, cells were treated with or without 50 μM NCL1 for 3 or 24h, and samples were pre-fixed with 2.5% glutaraldehyde in 0.1M phosphate buffer (pH 7.4) at 4°C. After fixation the specimens were post-fixed with 1% osmium tetroxide in 0.1M phosphate buffer (pH 7.4) for 45 min. They were subsequently dehydrated in a graded series of ethanol and embedded in epoxy resin. Ultra-thin sections were cut using an ULTRACUT-**S** (LEICA, Wetzlar, Germany) with a diamond knife, and stained with 2% uranyl acetate in distilled water for 15 min followed by a lead staining solution for 5 min. Sections were examined with a JEM-1011J (JEOL, Tokyo, Japan) electron microscope at 80KV.

### Fluorescent immunocytochemistry analysis

LNCaP cells were incubated with or without 40 μM CQ, and with or without 50 μM NCL1 for 24h, fixed with 4% formaldehyde for 10 minutes at room temperature and blocked with PBS containing 10% goat serum, 0.3 M glycine, 1% BSA and 0.1% tween-20 for 2h at room temperature. Staining of the treated cells with anti-LC3-II antibody was performed overnight at 4°C in PBS containing 1% BSA and 0.1% tween-20. An anti-rabbit polyclonal antibody (Abcam) at 1/250 dilution was used as the secondary antibody. Nuclei were counterstained with DAPI and shown in blue. Fluorescence images were observed and collected using A1 RSi multifunctional microscopy (Nikon, Tokyo, Japan) and analysis software.

### *In vivo* studies using a subcutaneous PCai1 model

Six-week-old male KSN/nu-nu nude mice were obtained from Nippon SLC and maintained as previously described [42]. PCai1 cells were cultured in T-75 flasks to confluence, trypsinized, and enumerated. Under isoflurane anesthesia 1 × 10^6^ PCai1 cells resuspended in 100 μL serum-free DMEM were injected subcutaneously into the dorsal side of the lumber vertebrae of mice without using matrigel. Then, intraperitoneal injection of vehicle containing DMSO that was equal in concentration to 1.0 mg/kg NCL1 (n=10), 10 mg/kg PCPA (n=5), or 0.5 mg/kg (n=10) or 1.0 mg/kg (n=10) NCL1 was performed 2 times per week. The amount of drug administration was determined on the basis of the *in vitro* WST-8 study. If we assume that NCL1 was uniformly distributed in the body, 1.0 mg/kg NCL1 would correspond to about 2.1 μM which was similar to the IC50 of NCL1 in a previous report [19]. Intraperitoneal injection started 4 days after tumor inoculation. Tumor size (determined by caliper measurement) and body weight were measured 2 times per week, and mice were sacrificed 9 weeks after the injection of cells.

All animal experiments were performed under protocols approved by the Institutional Animal Care and Use Committee of Nagoya City University School of Medical Sciences, and the approval number was H24M-58.

### Tissue array of human prostatectomy specimens

Prostatectomy specimens were obtained from the Aichi Cancer Center Hospital between 2009 and 2010. All specimens were obtained after the patients had provided written informed consent for the use of their tissues, according to an Institutional Review Board-approved protocol, and the approval number was NCU-6. All cases were reevaluated by a panel of experienced pathologists and rescored according to the Gleason grading system. Tissue arrays were prepared from formalin-fixed, paraffin-embedded tissue specimens of 46 prostate cancer patients.

### Immunohistochemical analysis

Deparaffinized tissue arrays were incubated with 1:200 diluted anti-LSD1 (Cell Signaling), and deparaffinized animal tissues were incubated with 1:100 diluted anti-CD31 (Santa Cruz). Antibody binding was visualized by a conventional immunostaining method, which was described previously, using an autoimmunostaining apparatus (HX System, Ventana, Tucson, AZ).

The intensity score of LSD1 expression was evaluated in normal prostate glands and carcinoma cores in each patient. For intensity of nuclear LSD1 immunoreactivity, the raw nuclear intensity data for luminal cells in normal prostate glands and tumor cells in prostate cancer cores were evaluated according to the Gleason pattern (Gleason pattern 3: n=25, Gleason pattern 4: n=15, Gleason pattern 5: n=6) using BZ-9000 multifunctional microscopy and analysis software (Keyence Japan, Osaka, Japan). The evaluations were repeated 5 times in each patient and the average intensity score in each core was calculated.

### TUNEL assay

Apoptotic cells in deparaffinized tissues were detected by terminal deoxy nucleotidyl transferase-mediated dUTP nick end labeling (TUNEL) assay performed using an In situ Apoptosis Detection Kit from Takara (Otsu, Japan) as per the manufacturer's protocol. Five randomly selected microscopic fields in each group were used to calculate the relative ratio of TUNEL-positive cells.

### Statistical analysis

Associations between different variables were assessed by Student's t test, ANOVA test, and Krusskal-Wallis test when appropriate. A value of *P*<0.05 was considered statistically significant.
